# Flame Retardancy of Epoxy Resins Modified with Few-Layer Black Phosphorus

**DOI:** 10.3390/polym15071655

**Published:** 2023-03-27

**Authors:** Yongzheng Zhao, Yan Li, Jiaxuan Li, Yifan Xiao, Wenmin Mu, Zhongwei Wang, Liang Song, Jinhong Yu

**Affiliations:** 1 College of Materials Science and Engineering, Shandong University of Science and Technology, Qingdao 266590, China; 2 College of Biology and Chemical Engineering, Qingdao Technical College, Qingdao 266555, China; 3 Key Laboratory of Marine Materials and Related Technologies, Zhejiang Key Laboratory of Marine Materials and Protective Technologies, Ningbo Institute of Materials Technology and Engineering, Chinese Academy of Sciences, Ningbo 315201, China

**Keywords:** few-layer black phosphorus, flame retardant, epoxy resin

## Abstract

Few-layer black phosphorus (BP)- and red phosphorus (RP)-modified diglycidyl ether of bisphenol A-based epoxy resins (EP) was prepared with 4,4′-diaminodiphenylsulfone as a curing agent. The thermal stability and flame-retardant properties of the modified EPs were compared. Both BP and RP were able to improve the flame-retardant properties of EPs, while the BP showed higher flame-retardant efficiency than RP. As a two-dimensional nanomaterial, BP exhibited good compatibility, high flame-retardant efficiency, and negligible impact on the mechanical and thermal stability of EP. Pyrolysis-gas Fourier-transform infrared spectroscopic analysis of EP showed that the addition of BP significantly inhibited the release of pyrolysis products in the gas phase. The modes of action for flame-retardant BPs in gas phase and condensed phase were proposed.

## 1. Introduction

Due to its advantages in terms of chemical, physical and mechanical properties, epoxy resin is applied in many areas, such as construction [1], adhesives [2], protective coatings [3], and electrical and electronic applications [4]. Since flame retardancy is always required in most application areas, flame-retardant epoxy has become a hot topic in both enterprises and research institutions. It is especially challenging to achieve flame retardancy in epoxy systems cured with aliphatic hardeners, owing to their high flammability. So far, halogen-containing [5] and phosphorus-containing [6] flame retardants are two kinds of highly effective flame retardants for epoxy resins. However, the application of halogen-containing flame retardants has been restricted by environmental legislation during the last twenty years. Therefore, there are many works on the structural design, synthesis and application of phosphorus-containing flame retardants for epoxy resins.

The reported phosphorus-containing flame retardants for epoxy resins including dialkylphosphinates (OP) [7], hexphenoxycyclotriphosphazene (HPCTP) [8] and other derivatives of hexachlorocyclotriphosphazene (HCCTP) [9], 9,10-dihydro-9-oxa-10-phos- phaphenanthrene-10-oxide (DOPO) [10,11,12,13] and its derivatives [14,15,16,17], diphenylphosphine oxide (DPO) [18] and its derivatives [15,19,20], 2,8-dimethylphenoxaphosphin-10-oxide (DPPO) and its derivatives [21], ammonium polyphosphate (APP) [22,23], etc. These flame retardants are used either via a reactive method or an additive method. Among them, DOPO, OP series, HPCTP, and DPO are popular commercial products for epoxy resins. DOPO and its derivatives dominate the phosphorus flame retardant market in the electronics and semiconductor packaging fields. However, a large dosage is required due to the small proportion of phosphorus in their molecular structure, leading to a decrease in their mechanical properties. Meanwhile, the use of DOPO also results in a detrimental impact on the glass transition temperatures of epoxy systems.

Compared with other phosphorous-containing organic compounds, red phosphorus (RP) is also a widely used flame retardant in many fields [24]. However, RP exhibits poor processability and compatibility with polymers. Moreover, because red phosphorus can easily absorb moisture, it needs to be used after coating, which leads to complexity in the application process.

Black phosphorus (BP) is an allotrope of RP. Like graphite, BP has a lamellar structure. Few-layer black phosphorus (BPs), also known as phosphene, can be obtained by exfoliating bulk BP [25,26]. As a novel two-dimensional nanomaterial, BPs has attracted considerable attention from researchers in recent years [27,28]. Due to the unique physical and chemical properties, the research on BPs in the fields of electronic devices, optoelectronic devices, and battery materials has always been a hot research topic [29]. As a flame retardant, BPs has the advantages of facile preparation (no complex synthesis involved and no coating required) and good compatibility with polymer matrix. The use of BPs as flame retardants for polyurethane and polyurethane acrylate has been reported by Xing et al. [30,31,32,33]. In addition, polyphosphazene-functionalized BP (BP-PZN) was incorporated into epoxy resin to study its flame-retardant properties [34]. The results showed that introduction of 2.0 wt.% BP-PZN in the epoxy resin leads to a 59.4% decrease in peak heat release rate and 63.6% decrease in total heat release. Although the BP-PZN exhibited significantly improved dispersibility of the phosphorene and excellent flame retardancy, evaluation of the role of BPs is difficult due to the coexistence of the polyphosphazene, which is also a flame retardant. In another work, melamine-formaldehyde (MF) was employed to functionalize BPs by Wu et al. [35], and BP@MF was used as a flame retardant for DDM-cured DGEBA epoxy resin. The introduction of BP@MF significantly improved the flame retardancy of the epoxy resin. The LOI value of epoxy resin containing 1.2 wt.% BP@MF increased to 31.1, reaching a UL-94 V-0 rating. Moreover, the performance of BP@MF was compared with MF and BPs. Zou et al. [36] prepared a black phosphorous and carbon nanotube (MCNTs) nanohybrid (BP-MCNTs) and used it for epoxy resin flame retardant. The peak of heat release rate and total heat release of epoxy nanocomposites reduced by 55.81% and 41.17% at a phosphorus content of only 1 wt.%.

Here, we report the facile synthesis of BP nanosheets and an investigation of the thermal stability, mechanical property, flame retardancy and fire behavior of DGEBA epoxy resins modified with BP nanosheets. BP nanosheets were compared with RP as a flame retardant for amine-cured epoxy resins. The structure of BP and morphology of char residues were characterized with scanning electron microscopy (SEM). The mode of action for flame retardant of EP/BP was revealed by cone calorimeter (CCT) and thermogravimetric analysis coupled with infrared spectrometry (TG-IR). According to the results, EP modified with BP has better flame retardancy, which makes it a promising for application for flame retardancy.

## 2. Experimental Section

### 2.1. Materials

Unless stated otherwise, all raw materials were used as received. The bisphenol-A-type epoxy resin (DGEBA, trade code: E-44) with an epoxy value of 0.44 was purchased from Zhenjiang Danbao Resin Co., Ltd. (Jiangsu, China). The red phosphorus (RP), 4,4′-diaminodiphenyl sulfone (DDS) and N-methyl pyrrolidone (NMP) were purchased from Aladdin Industrial Corporation. (Shanghai, China). The black phosphorus (BP) was supplied by Sino-Linchem Group (Guangxi, China).

### 2.2. Preparation of Few-Layer BPs

Few-layer BPs was prepared using the liquid phase exfoliation (LPE) method [26]. LPE is used as the most feasible tool for the large-scale production of few-layer 2D materials, providing high-quality dispersions of 2D materials dispersed in solvents. We used the liquid exfoliation method of black phosphorus in N-methyl-2-pyrrolidone (NMP) to form few-layer phosphorene nanosheets. NMP is a suitable solvent for BP exfoliation due to its high boiling point and excellent surface tension. First, bulk BP was ground into small particles and mixed with NMP; then, the BP/NMP mixture was sonicated for 24 h in an ice bath. After ultrasonic dispersion, the resulting solution was centrifuged for 1 h at 5000 rpm to obtain supernatant containing BPs, which was further centrifuged for 30 min at 10,000 rpm. Finally, the collected precipitate was dried in a vacuum oven to obtain few-layer BPs.

### 2.3. Preparation of BP-Modified Epoxy Resins

The preparation procedure of BP-modified epoxy resins was as follows: the desired amount of BPs was dispersed in 10 mL ethanol and sonicated for 30 min. Then, 30 g preheated epoxy resin was added to the treated ethanol dispersion. The mixture was then heated at 150 °C and mechanically stirred for 1 h to remove the ethanol and evenly disperse the BPs. Afterwards, 8.57 g of DDS powder was added to the above mixture and stirring was continued for 2 min. Finally, the processed resin sample was poured into a mold and placed in an oven (preheated to 120 °C). After 2h, the oven was heated to 180 °C (heating rate of 5 °C/min) and then held at 180 °C for 2 h. At the end of the curing process, the resin samples were slowly cooled to room temperature to prevent cracking. 

For comparison purposes, RP was incorporated in epoxy resin using the same procedure. For convenience, the epoxy resins containing BP and RP were denoted as EP/BP-x and EP/RP-x, respectively, where x represents the weight percent of P in the sample. EP/P-0 denotes the cured epoxy resin without BP or RP.

### 2.4. Characterization

The microstructure of BP, the cross-section of EP/BP and the microstructure of carbon residues of EP/BP samples were analyzed by scanning electron microscopy (SEM, Nova Nano SEM450, Hillsborough, USA) operating at an accelerating voltage of 10 kV. The corresponding elemental composition was investigated using energy-dispersive X-ray spectrometry (EDS). The microstructure of the BPs was analyzed by transmission electron microscopy (TEM, JEOL JEM-2100F, Osaka, Japan). The surface topography of BPs was characterized by atomic force microscopy (AFM, Bruker, Karlsruhe, Germany). X-ray diffractometry (XRD, Rigaku D/MAX 2500 PC, Tokyo, Japan) was performed to determine the crystal structures of the samples. Raman spectroscopy (Horiba Lab-RAM HR Evolution, Tokyo, Japan) was performed under an excitation of 514 nm argon laser to observe the bulk BP, few-layer BPs and char layer structure of EP composites. 

LOI measurements were conducted on an oxygen index instrument (BinGo Instrument Co., Ltd., Shenzhen, China) according to the ISO4589-2017 standard with a specimen size of 130 × 6.5 × 3 mm^3^. According to UL-94 test standard, the vertical burning test (UL-94 test) was performed using a BG5210 instrument (BinGo Instrument Co., Ltd., Shenzhen, China) with a specimen size of 130 × 13 × 3 mm^3^. The fire behavior was characterized using a cone calorimeter (CCT, East Grinstead FTT, Sussex County, UK) based on the ISO 5660 standard at an external heat flux of 50 kW/m^2^ and a distance between the sample and the cone of 25 mm. The sample dimensions were 100 × 100 × 3 mm^3^. X-ray photoelectron spectroscopy (XPS, Thermo Fisher Escalab 250Xi, Massachusetts, USA) was performed to analyze the element composition of the residues after the CCT test. 

Dynamic mechanical analysis (DMA, Hitachi DMS6100, Tokyo, Japan) was employed to test the storage modulus and tan δ of EP, EP/BP and EP/RP composites. The glass transition temperature (*T*_g_) of EP nanocomposites can be measured using the tan δ curves of DMA, and the peak value of tan δ reflects the *T*_g_ of the nanocomposites. DMA was performed under air using the double cantilever clip mode from 100 to 200 °C at a heating rate of 5 °C/min, with a frequency of 1 Hz and an amplitude of 3 μm. The sample dimensions were 35 × 6.5 × 3 mm^3^. Crosslinking density ($\nu_{e}$) was calculated according to the method presented in the literature [37]. 

Thermogravimetric analysis (TGA, Netzsch TG-209F3, Bavaria, Germany) was performed to analyze the thermal stability of EP composites. A sample powder of 5–10 mg was heated from 100 to 700 °C under nitrogen, applying a heating rate of 10 °C/min and a flow rate of 50 mL/min. Thermogravimetric analysis coupled with infrared spectrometry (TG-IR) was carried out on a TG209F3/Tensor 27 thermo-analyzer instrument combined with a Bruker spectrometer from 20 to 800 °C at 10 °C/min (N_2_ atmosphere, flow rate of 100 mL/min). 

## 3. Results and Discussion

### 3.1. Characterization of BPs 

**Figure 1:**
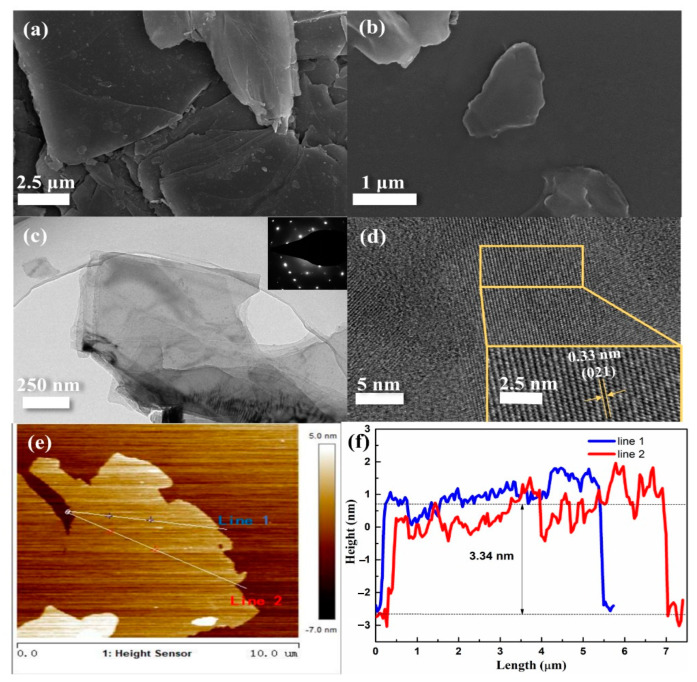
SEM images of bulk BP (**a**) and BPs (**b**); TEM image and SAED pattern of BPs (**c**); HRTEM image of BPs (**d**); AFM image of BPs (**e**); the height of line 1 and line 2 (**f**).

Figure 1a shows the typical multilayer structure of bulk BP [27,34]. After exfoliation, the BP nanosheets became thinner, with a length of about 3 μm and a width of about 2.5 μm, as shown in Figure 1b. Transmission electron microscopy (TEM) is usually used to observe the fine internal structure of the sample. Figure 1c shows the TEM and selected area electron diffraction (SAED) pattern of the as-prepared few-layer BPs. The TEM image shows that the BPs was successfully exfoliated. The SAED pattern shows the orthorhombic crystalline structure of the BPs. Lattice spacing in the HRTEM image was calculated using Digital Micrograph software. Figure 1d presents the high-resolution TEM (HRTEM) image of the BP, where the lattice fringes of 0.33 nm are assigned to the (021) plane of BP [27], which is in line with the results of the XRD tests. Few-layer BPs is also evidenced by the atomic force microscopy (AFM) images in Figure 1e,f. The AFM images present BP as having a relatively uniform surface, with an average thickness of 3.34 nm. According to the literature [38], 0.53 nm is generally considered to be the thickness of a single layer of phosphorene. Therefore, the BPs with an average thickness of 3.34 nm observed in Figure 1e is composed of approximately six or seven layers of phosphorene; these results are highly consistent with the observations in Figure 1b,d, demonstrating that few-layer BPs was successfully fabricated. 

**Figure 2:**
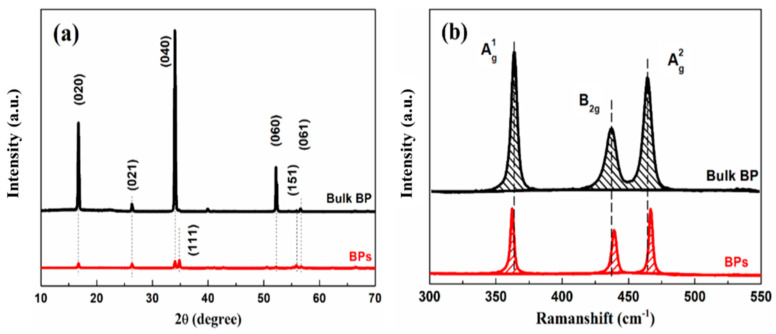
XRD patterns (**a**) and Raman spectra (**b**) of bulk BP and BPs.

The XRD patterns and Raman spectra of bulk BP and BPs are shown in Figure 2a,b. For bulk BP, diffraction peaks were observed at 16.8°, 26.6°, 34.2°, 52.4°, 56.2° and 56.8°, corresponding to the (020), (021), (040), (060), (151) and (061) planes, respectively [39]. The BPs exhibited all of the diffraction peaks that could be observed in bulk BP; however, the intensities of all of the diffraction peaks were weaker, which can be attributed to the thinning of the molecular layer after exfoliation [34]. In addition, in the BPs spectrum, the intensity of the (111) plane was higher than that of the (040) plane. This phenomenon was contrary to what was observed in bulk BP, because the stacking of the interlayered planes is affected by the weakening of van der Waals interactions between the phosphorene layers [27]. The Raman spectrum of bulk BP showed three characteristic peaks located at about 362.8, 437.2 and 463.8 cm^−1^, which could be indexed as $A_{g}^{1}$, $B_{2g}$ and $A_{g}^{2}$ vibrational modes of BP, respectively [27]. Although the same peaks were also detected in BPs, the peak position of $A_{g}^{1}$ model for BPs was more biased towards a lower wavenumber relative to bulk BPs. Meanwhile, the peak position of the $A_{g}^{2}$ model was biased towards high wavenumbers. This phenomenon was caused by a decrease in the number of black phosphorus layers [40]. 

### 3.2. Dispersibility of BPs and RP in Epoxy Composites

**Figure 3:**
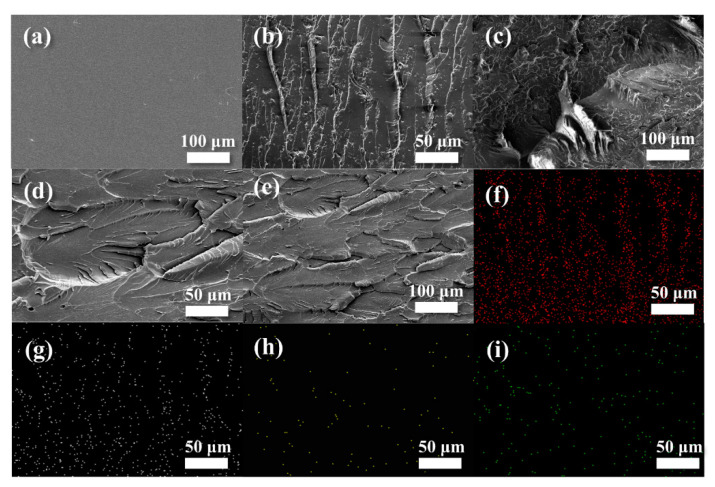
SEM images of the section of EP/P-0 (**a**), EP/BP-0.9 (**b**,**c**) and EP/RP-0.9 (**d**,**e**); EDS mapping of C (**f**), O (**g**), N (**h**) and P (**i**) elements for the same section of the image (**b**).

To characterize the dispersion of fillers in EP composites and the effect of filler on the texture of the matrix, the sections of EP/P-0, EP/RP-0.9 and EP/BP-0.9 were characterized by SEM and EDS. Figure 3a shows that the fracture surface of EP/P-0 was very smooth. The section of EP/RP-0.9 had more irregular cracks, while the section of EP/BP-0.9 had slight undulations and cracks (Figure 3b–e). This indicates that the addition of RP and BPs had a certain effect on the texture of the epoxy matrix, and the influence of BPs on the matrix was smaller than that of RP. The epoxy composites show a rough fracture surface due to the matrix shear yielding or local polymer deformation. Therefore, the epoxy composites failed in a brittle manner due to the mode of action for crack deflection induced by the nanosheets. Figure 3f–i present the elemental surface mapping with energy dispersive spectroscopy (EDS) in the region of Figure 3b, revealing the distributions of C, O, N and P elements within the EP/BP-0.9 composites. As shown in Figure 3i, the phosphorus element was evenly distributed in the matrix, indicating that BPs did not re-aggregate in the epoxy matrix.

### 3.3. Flame-Retardant Properties 

**Figure 4:**
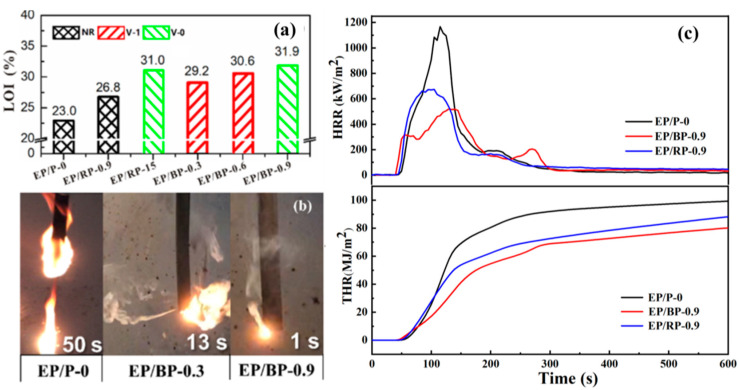
LOI values and UL-94 vertical burning of epoxy resins (**a**); video screenshots of EP/P-0, EP/BP-0.3 and EP/BP-0.9 during UL-94 test (**b**); HRR curves and THR curves (**c**) of EP/P-0 and EP/BP-0.9.

The flame retardancy of the epoxy resin samples was evaluated by LOI test and UL-94 vertical combustion test. The obtained results are shown in Figure 4a. EP/P-0 showed high flamability with a low LOI value of 23.0%. Whereas the LOI values of EP/BP samples increased apparently with the introduction of BPs. Adding only 0.3 wt.% BPs, the LOI value of the epoxy resin increased to 29.2%. Compared with BPs, the flame retardancy efficiency of RP was fairly low. The LOI value of EP/RP-15 (30.0%) was lower than that of EP/BP-0.9 (31.9%). According to the UL-94 test results, EP/BP-0.3 and EP/BP-0.6 reached a UL-94 V-1 rating and EP/BP-0.9 reached a V-0 rating. The EP/RP-0.9 showed no rating, and EP/RP required a phosphorus content of 15% to achieve the V-0 rating. In summary, whether RP or BPs is introduced, the flame retardancy of epoxy resins will be improved. The flame retardancy performance of BP-modified epoxy resins is much better than that of RP-modified ones. 

Figure 4b shows some screenshots of the UL-94 test videos for EP/P-0, EP/BP-0.3 and EP/BP-0.9, respectively. The combustion process of EP/P-0 was smooth, and it was accompanied by dripping phenomenon when burning. Although EP/BP-0.3 did not reach a V-0 rating due to small amount of BPs added, the blowout effect was clearly observed during the combustion process: while one or more visible smoke streams were blowing out of the sample, the flame in the blowing direction was significantly reduced compared to the flame in the opposite direction. As for the EP/BP-0.9, the combustion process and extinguish process were both rapid. Its blowout effect was also rapid and efficient.

The fire behaviors of EP/P-0, EP/BP-0.9 and EP/RP-0.9 were tested with CCT. The HRR and THR curves of EP/P-0, EP/BP-0.9 and EP/RP-0.9 are presented in Figure 4c and detailed data are presented in Table 1. The results indicate that EP/P-0 exhibited high flammability with PHRR and THR values of 1166.7 kW/m^2^ and 99.3 MJ/m^2^, respectively. The PHRR and THR of EP/RP-0.9 are 673.9 kW/m^2^ and 88.2 MJ/m^2^, which were 57.8% and 88.8% of EP/P-0, respectively. Addition of 0.9 wt.% BPs led to the significant increase in t_PHRR_ (113% of EP/P-0) and the decreases in PHRR (44.6% of EP/P-0) and THR (80.8% of EP/P-0). This phenomenon indicates that the addition of BPs slows down the heat release rate of the EP/BP nanocomposites during the burning process, thereby improving the flame retardancy of the epoxy composites. 

Filler efficiency is often used to characterize the contribution per unit weight of filler to flame-retardant properties. The filler efficiencies for PHRR, LOI and THR are defined as *φ*_PHRR_, *φ*_LOI_, and *φ*_THR_ according to Equations (1)–(3) [35], respectively. The effect of BPs as flame retardants was compared with those described in the literature. A comparison of the results is provided in Table 2. It is clear that with the lowest amount of FRs, the BPs used in our study exhibited the highest value of *φ*_LOI_, *φ*_PHRR_ and *φ*_THR_. This indicates that BPs has an excellent effect on the enhancement of flame retardancy in epoxy composites.

(1) $$\varphi \mathrm{PHRR}=|\frac{\mathrm{PHRR}-\mathrm{PHRRm}}{\mathrm{PHRRm}\times \mathrm{wt}.\%}|$$

(2) $$\varphi \mathrm{LOI}=|\frac{\mathrm{LOI}-\mathrm{LOIm}}{\mathrm{LOIm}\times \mathrm{wt}\%}|$$

(3) $$\varphi \mathrm{THR}=|\frac{\mathrm{THR}-\mathrm{THRm}}{\mathrm{THRm}\times \mathrm{wt}\%}|$$

Abbreviations: PHRR is peak heat release rate of the composite, and PHRR_m_ is peak heat release rate of the matrix material. LOI is limiting oxygen index of the composite, and LOI_m_ is limiting oxygen index of the matrix material. THR is total heat release of the composite, and THR_m_ is total heat release of the matrix material. In addition, wt.% is the weight fraction of filler. 

In order to better understand the mode of action for flame retardancy of BPs in epoxy, the flame inhibition effect, charring effect and formation of a protective barrier layer effect of BPs were quantitatively evaluated. According to Equations (4)–(6) [41,42], the values of the three flame retardant modes were calculated, and the results are shown in Table 3.

(4) $$\mathrm{Flame}\;\mathrm{inhibition}=1-\frac{{\mathrm{EHC}}_{\mathrm{EP}/\mathrm{BP}-0.9}}{{\mathrm{EHC}}_{\mathrm{EP}}}$$

(5) $$\mathrm{Charring}=1-\frac{{\mathrm{TML}}_{\mathrm{EP}/\mathrm{BP}-0.9}}{{\mathrm{TML}}_{\mathrm{EP}}}$$

(6) Barrier and protective layer=1-PHRREP/BP-0.9PHRREPTHREP/BP-0.9THREP

According to the data presented in Table 3, the barrier and protective effect (44.73%) was the main flame-retardant mode of BPs in epoxy resins, accompanied by flame inhibition effect and charring effect. The barrier and protective effect mainly come from (1) the high thermal stability and nanosheets structure of BPs; (2) the catalytic effect on the formation of carbon layer. This plays an important role in reducing heat transfer and mass exchange during combustion. The improved flame inhibition effect is attributed to the release of a large number of free radicals (PO_2_•, PO•, and HPO•), which can quench free radical reaction by capturing H• and OH• [34]. 

**Figure 5:**
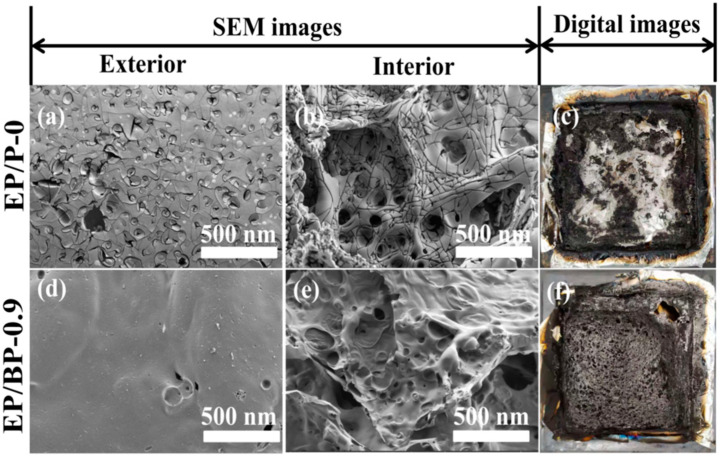
SEM images of external surface and internal surface of the char residues after CCT tests for EP/P-0 (**a,b**) and EP/BP-0.9 (**d,e**); digital photos of external char residues after CCT tests for EP/P-0 (**c**) and EP/BP-0.9 (**f**).

To investigate the mode of action for flame retardant of BPs in the condensed phase, SEM, Raman and XPS tests were performed on the residual carbon obtained from CCT. Figure 5 shows the digital photos and SEM images of residues after the CCT of EP/P-0 and EP/BP-0.9. Compared with EP/P-0, the addition of BPs led to the formation of a continuous carbon layer, which apparently increased the amount of char residue. This result is consistent with the results of CCT, whereby the TML for EP/BP-0.9 decreased ca.10% compared with EP/P-0. The external and internal surface of the residues of EP/P-0 was full of holes and cracks, while those of the residues of EP/BP-0.9 were relatively smooth, with only a few pores. The formation of such continuous and dense layer was attributed to the catalytic effect of BPs. This layer inhibits the transfer of heat and oxygen and protects the polymer matrix during combustion. These results are consistent with those presented in Table 1 and Table 3, further confirming the remarkable effect of BPs as flame retardant for EP.

**Figure 6:**
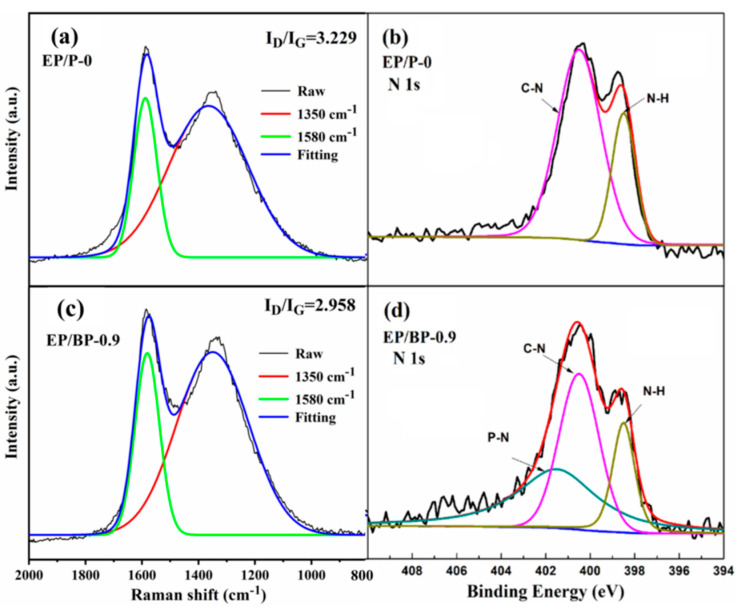
Raman spectra of the char residues after CCT tests for EP/P-0 (**a**) and EP/BP-0.9 (**c**); the high-resolution N*1s* XPS spectra of EP/P-0 (**b**) and EP/BP-0.9 (**d**).

**Figure 7:**
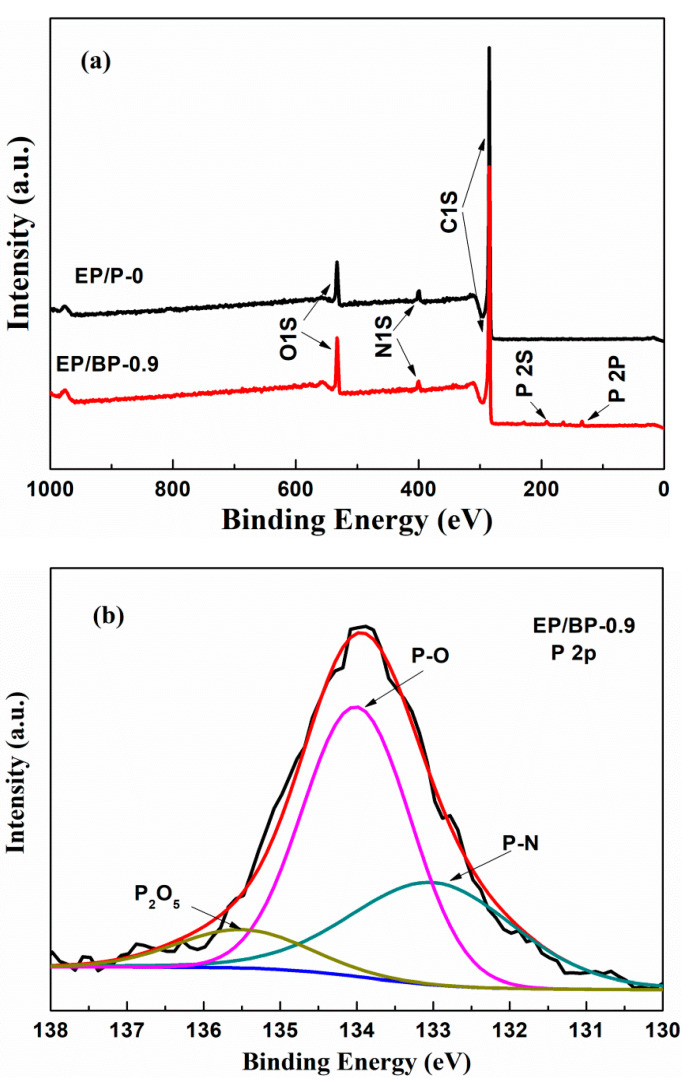
XPS survey spectra of the residual char (**a**); high-resolution P *2p* XPS spectra of EP/BP-0.9 (**b**).

Figure 6 depicts the Raman spectra and the high-resolution N*1s* XPS spectra of the char residues of EP/P-0 and EP/BP-0.9 after the cone calorimetry test. The characteristic absorption peak at 1350 cm^−1^ was the D peak, representing the disordered carbon structure, and the characteristic absorption peak at 1582 cm^−1^ was the G peak, representing the ordered carbon structure. The ratio of the intensity of the D peak to the G peak (*I*_D_/*I*_G_) represented the degree of graphitization of the residual carbon. The lower the ratio, the higher the degree of graphitization [43]. The *I*_D_/*I*_G_ ratio of EP/P-0 was 3.229, which dropped to 2.958 after the addition of BPs. This indicates that the addition of BPs was beneficial to the formation of an ordered carbon structure in the residue [44]. This effect promotes the formation of a stable and continuous carbon layer. This result is consistent with those reported for CCT and SEM, confirming the role of BPs in improving the flame retardancy of EP. According to the XPS results (Figure 7a), the char residues of EP/P-0 and EP/BP-0.9 both contain C, N, and O elements, while the P element appears in EP/BP-0.9 due to the addition of BPs. The high-resolution XPS spectra of EP/BP-0.9 in the N*1s* region (Figure 6d) consisted of three peaks (401.8, 400.6 and 398.5 eV) corresponding to P-N, C-N, and N-H bonds, respectively. C-N and N-H bonds originated from the reaction of EP with DDS, which appeared in N1s XPS spectra for EP/P-0 (Figure 6b). P-N bonds were formed by the combination of BPs and the N element in the curing agent DDS. This result revealed the formation of phosphorus–nitrogen oxide network and was confirmed by the high-resolution P *2p* XPS spectra of EP/BP-0.9 (Figure 7b). This phosphorus–nitrogen oxide network indicates that BPs take part in the formation of compact and dense carbon layers. This is consistent with the SEM results of the residue (Figure 5). The phosphorus-containing flame retardants mentioned above have good flame-retardant performance in the polymer matrix containing nitrogen elements. The effect and mode of action for P-N synergy have also been reported previously [45]. Therefore, we believe that in the epoxy cured by the N-containing curing agent DDS, composite materials with good flame-retardant properties can be obtained by simply introducing black phosphorus-containing phosphorus elements.

### 3.4. Thermal Properties

**Figure 8:**
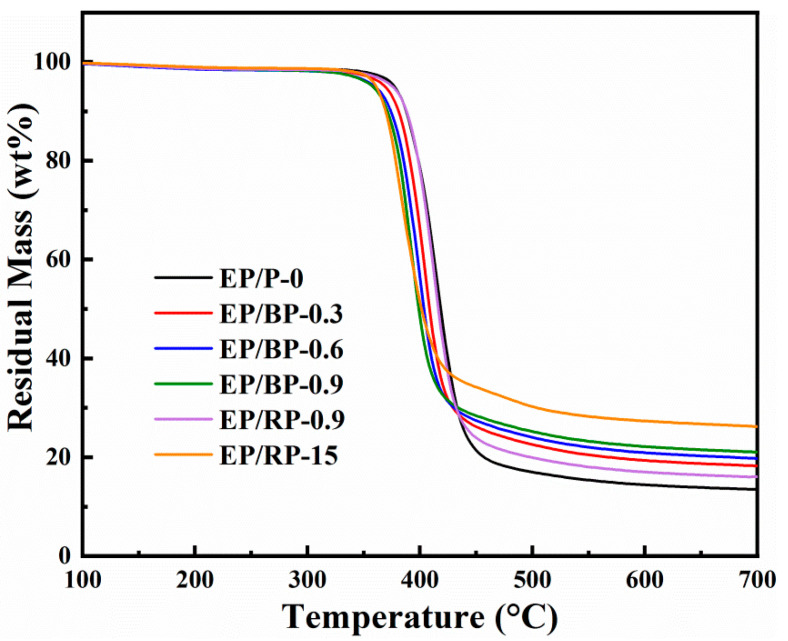
TG curves of epoxy resins under N_2_.

The thermal stability of the epoxy resins was investigated by TGA. The TG curves of the epoxy composites under nitrogen are shown in Figure 8. Table 4 lists the selected data of 5 wt.% mass loss temperature (*T*_5%_) and the char yield at 700 °C.

Figure 8 shows that the weight loss behaviors of all flame-retardant epoxy composites were similar to that of EP/P-0. Under N_2_ atmosphere, whether BPs or RP were used as additives, the *T*_5%_ of all modified samples were all lower than that of EP/P-0, and gradually decreased with increasing phosphorus content. This phenomenon may be attributed to the catalytic effect of BPs or RP on the decomposition of epoxy matrix. This catalytic effect leads to the formation of carbon layers, which protect the EP and increase the amount of char residue. The residue mass percentage of EP/BP-0.9 reached 21.1%, which was 157.5% of EP/P-0. The residual mass percentage of EP/RP-0.9 was 16.0%, which was only 2.6% higher than that of EP/P-0. Obviously, the effect of BPs is more obvious than that of RP. It is a fact that BPs is uniformly dispersed in epoxy as two-dimensional nanomaterials, and can act as templates and skeletons for the formation of multiple and overlapping char via the so-called “labyrinth effect” [46]. 

The *T*_g_ values, storage modulus and crosslinking density (*ν_e_*) of the epoxy resins were measured by DMA. The data are listed in Table 4. The storage modulus curves and tan δ curves are shown in the Supplementary Materials (Figure S2). As for EP/BP, the *T*_g_ value increased with increasing P content. When P content was 0.9 wt.%, the *T*_g_ was ca. 8 °C higher than EP/P-0. This phenomenon can be attributed to the high stiffness and sheet-like structure of BPs restricting the movement of molecular chains in the matrix [47]. This effect also influenced the *ν_e_* of EP, resulting in the decrease in *ν_e_* value. As for EP/RP composites, the *T*_g_ was almost unchanged when the P content was 0.9 wt.%; however, the storage modulus was higher than that of EP/P-0. This is attributed to the high stiffness of RP [34].

### 3.5. Mode of Action for Flame Retardancy 

**Figure 9:**
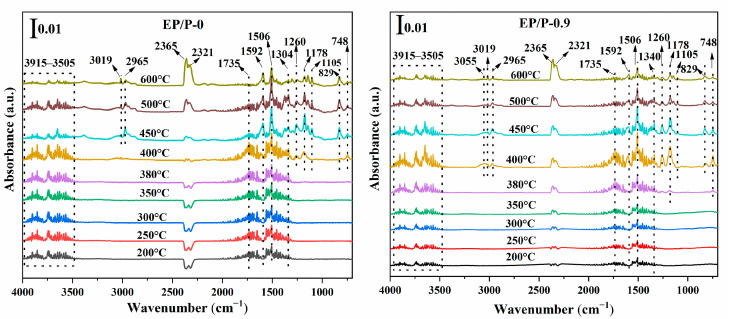
FTIR spectra of gas phase pyrolysis products at representative temperatures obtained from TG-FTIR testing.

To reveal the mode of action for the flame retardancy of EP/BP composites, TG-FTIR was employed to investigate the gas phase products of EP/P-0 and EP/BP-0.9. The FTIR spectra of gas phase-pyrolysis products of EP/P-0 and EP/BP-0.9 at different temperatures are shown in Figure 9. Hydrocarbons (3019 and 2965 cm^−1^), aromatic compounds (3055, 1592, 1506, 829 and 748 cm^−1^), phenol (3915~3505, 1260 cm^−1^), carbonyl compounds (1735 cm^−1^) and CO_2_ (2365 and 2321 cm^−1^) were observed for both samples. It should be noted that almost no aromatic compounds (3055 and 1592 cm^−1^) were detected above 450 °C for EP/BP-0.9. Moreover, the amount of CO_2_ released at high temperature decreased with the addition of BPs. This means that the addition of BPs inhibited combustion. The results were in agreement with those of CCT and SEM. The inhibition of combustion derives from the promotion of carbon layer formation by BPs.

**Figure 10:**
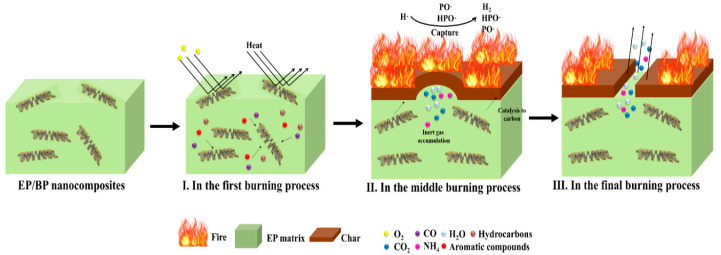
Mode of action for flame retardant of EP/BP composites.

Based on the above results and the analysis of the combustion behavior of EP/BP composites, a possible mode of action for flame retardancy was proposed, as shown in Figure 10. At the beginning of combustion, BPs inhibit the docomposition of EP due to a physical barrier effect (one of the main mode of action for flame retardant of layered nanomaterials [48]). During combustion, BPs form active radicals (PO• and HPO•), which can reduce the generation of combustible gases by trapping H• and OH• radicals, thereby achieving the effect of flame inhibition (Table 3). On the other hand, another part of the phosphorus in BPs is oxidized to phosphoxides and then react with water to generate a variety of phosphoric acid derivatives, which catalyze the charring of polymers in the condensed phase [49]. This results in the formation of a continous layer (Figure 5). This layer protects the matrix and reduces the amount of aromatic compounds and CO_2_ released (Table 1 and Figure 9).

## 4. Conclusions

In this work, few-layer BPs and RP were incorporated into epoxy resin, respectively, to prepare flame-retardant epoxy resins. Introduction of either RP or BPs can improve the flame retardancy of epoxy resins. BPs showed much higher flame retardancy efficiency than RP. Compared to EP/RP-0.9, EP/BP-0.9 was able to achieve UL-94 V-0 rating with LOI value of 31.9. Compared to neat EP, PHRR of EP/BP-0.9 and EP/RP-0.9 exhibited decreases of 55.4% and 42.2%, respectively. The THR of the EP/BP-0.9 and EP/RP-0.9 decreased by 19.2% and 11.2%, respectively. The introduction of both RP and BP showed negligible effects on the *T*_g_ value of EP; however, the *T*_g_ of EP/BP-0.9 (197 °C) was higher than EP/RP-0.9 (188 °C). TG-FTIR analysis demonstrated that all the intensities of pyrolysis products were inhibited with the introduction of BP. The improvement in the flame-retardant performance of EP/BP is mainly due to the combined effect of catalytic carbonization, physical barrier and free radical quenching of the BP nanosheets. 

## Figures and Tables

**Table 1:** Cone calorimetry test data of the EP/P-0 and EP/BP-0.9.

Sample	tPHRR (s)	PHRR (kW/m^2^)	THR (MJ/m^2^)	av-EHC (MJ/kg)	av-CO (kg/kg)	av-CO_2_ (kg/kg)	TML (wt.%)	TTI (s)	Char Residual (wt.%)
EP/P-0	115	1166.7	99.3	23.3	0.09	1.68	96.4	40	3.55
EP/BP-0.9	130	520.8	80.2	19.0	0.12	1.24	86.0	38	13.20
EP/RP-0.9	105	673.9	88.2	23.7	0.10	1.51	89.8	38	10.19

Abbreviations: t_PHRR_, time to peak heat release rate; PHRR, peak heat release rate; THR, total heat release; av-EHC, the average effective heats of combustion; av-CO, average carbon monoxide yields; av-CO_2_, the average carbon dioxide yields; TML, total mass loss; TTI, time to ignition.

**Table 2:** The efficiency of FRs in the literature and this work.

FRs	BP-Bulk	BP-PZN	BP	BP@MF	BPs
Fraction (wt.%)	2	2	1.2	1.2	0.9
*φ*_LOI_	-	-	5.1%	21.1%	43.0%
*φ*_PHRR_	24.4%	29.7%	32.8%	36.1%	61.5%
*φ*_THR_	21.8%	31.8%	2.3%	10.5%	21.4%
UL-94	V-0	-	NR	V-0	V-0
Matrix	E-44	E-44	E-51	E-51	E-44
Ref.	[34]		[31]		This work

**Table 3:** Quantitative assessment of the three main flame-retardant modes.

Sample	Flame Inhibition Effect	Charring Effect	Barrier and Protective Effect
EP/BP-0.9	18.45%	10.79%	44.73%

**Table 4:** Thermal properties data of epoxy thermosets.

Sample	*T*_5%_ (°C)	Char Yeild at 700 °C (wt.%)	*T*_g_ (°C)	Storage Modulus (MPa)		ν_e_ (×10^3^ mol/m^3^)
				50 °C	*T*_g_ + 40 °C	
EP/P-0	377.5	13.4	189	1331.87	10.36	1.59
EP/BP-0.3	367.2	18.2	186	1388.82	11.18	1.68
EP/BP-0.6	359.9	19.8	190	1141.44	9.48	1.42
EP/BP-0.9	357.6	21.1	197	1470.61	9.18	1.38
EP/RP-0.9	374.6	16.0	188	1579.31	12.80	1.92
EP/RP-15	359.9	26.2	191	1449.56	13.94	2.09

Note: Crosslink density (ν_e_) was calculated according to the literature^33^: ν_e_ = E’/3RT; where E’ is the storage modulus of cured EP at (*T*_g_ + 40) °C, T is the absolute temperature, and R is the gas constant.

## Data Availability

No new data were created or analyzed in this study. Data sharing is not applicable to this article.

## Supplementary Materials

The following supporting information can be downloaded at: https://www.mdpi.com/article/10.3390/polym15071655/s1. Figure S1: Video screenshots of EP/P-0, EP/BP-0.3 and EP/BP-0.9 during UL-94 testing; Figure S2: Storage modulus curves (a) and tan δ curves (b) of the epoxy thermosets.
