# Supplementary material for: Flame Retardancy of Epoxy Resins Modified with Few-Layer Black Phosphorus
Source: Polymers (Basel). 2023 Mar 27;15(7):1655. doi: 10.3390/polym15071655 (PMC10097328; doi:10.3390/polym15071655)
Supplement: Supplementary file 1 [file polymers-15-01655-s001.zip › polymers-2135767-supplementary.pdf]

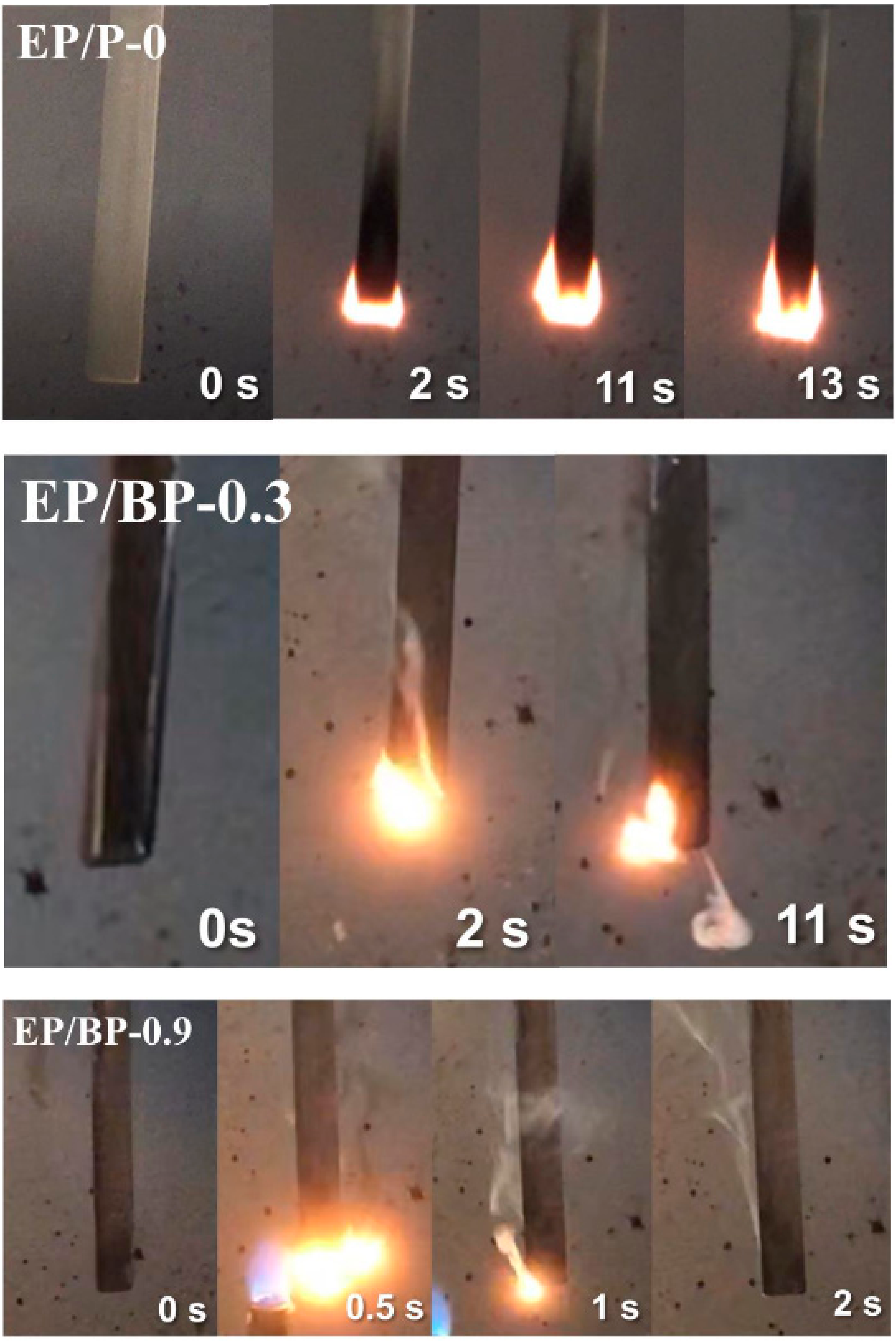

Figure S1. Video screenshots of EP/P-0, EP/BP-0.3 and EP/BP-0.9 during UL-94 testing.

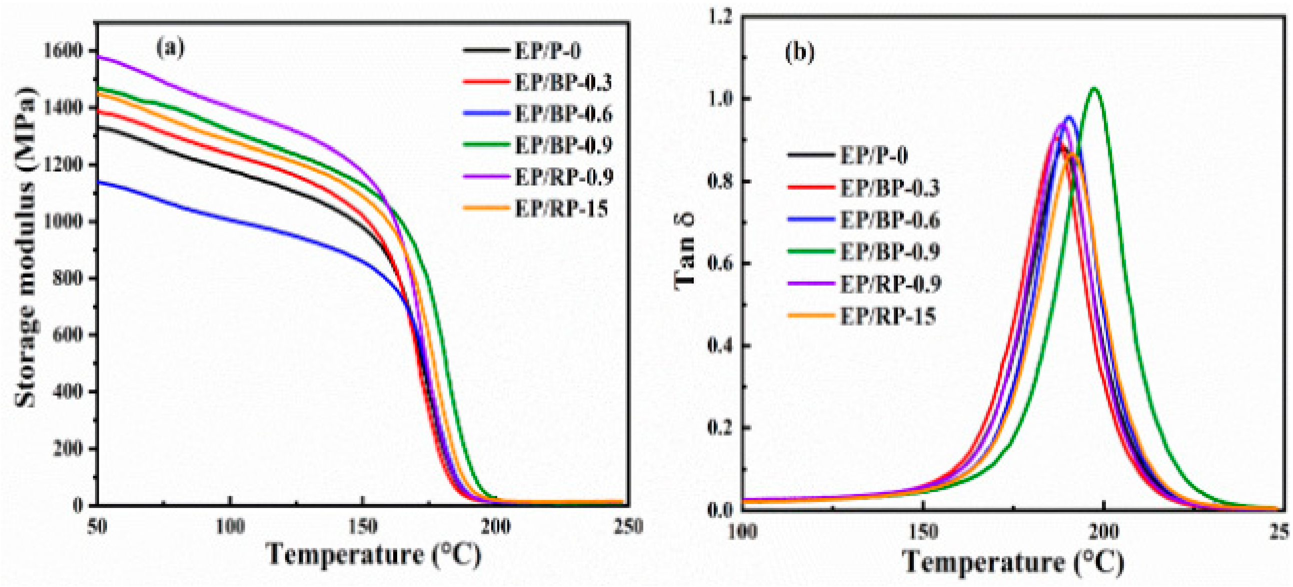

Figure S2. Storage modulus curves(a) and tan δ curves(b) of the epoxy thermosets.
